# Yoga for Treating Rheumatoid Arthritis: A Systematic Review and Meta-Analysis

**DOI:** 10.3389/fmed.2020.586665

**Published:** 2020-11-27

**Authors:** Xiangling Ye, Zehua Chen, Zhen Shen, Guocai Chen, Xuemeng Xu

**Affiliations:** ^1^The Fifth Clinical Medical College, Guangzhou University of Chinese Medicine, Guangzhou, China; ^2^Kunming Municipal Hospital of Traditional Chinese Medicine, The Third Affiliated Hospital of Yunnan University of Chinese Medicine, Kunming, China; ^3^Foshan Hospital of Traditional Chinese Medicine, Guangzhou University of Chinese Medicine, Guangzhou, China; ^4^Guangdong Second Traditional Chinese Medicine Hospital, Guangzhou, China

**Keywords:** yoga, non-pharmacologic strategies, rheumatoid arthritis, meta-analysis, review

## Abstract

**Purpose:** Rheumatoid arthritis (RA) is a pervasive inflammatory autoimmune disease that seriously impairs human health and requires more effective non-pharmacologic treatment approaches. This study aims to systematically review and evaluate the efficacy of yoga for patients with RA.

**Methods:** Medline (through PubMed), Cochrane Library, EMBASE (through SCOPUS), and Web of Science database were screened through for articles published until 20 July 2020. Randomized controlled trials (RCTs) of yoga in patients with RA were included. Outcomes measures were pain, physical function, disease activity, inflammatory cytokines, and grip strength. For each outcome, standardized mean differences (SMD) and 95% confidence intervals (CI) were calculated.

**Result:** Ten trials including 840 patients with RA aged 30–70 years were identified, with 86% female participants. Meta-analysis revealed a statistically significant overall effect in favor of yoga for physical function (HAQ-DI) (5 RCTs; SMD = −0.32, 95% CI −0.58 to −0.05, *I*^2^ = 15%, *P* = 0.02), disease activity (DAS-28) (4 RCTs; SMD = −0.38, 95% CI −0.71 to −0.06, *I*^2^ = 41%, *P* = 0.02) and grip strength (2 RCTs; SMD = 1.30, 95% CI 0.47–2.13, *I*^2^ = 63%, *P* = 0.002). No effects were found for pain, tender joints, swollen joints count or inflammatory cytokines (i.e., CRP, ESR, IL-6, and TNF-α).

**Summary:** The findings of this meta-analysis indicate that yoga may be beneficial for improving physical function, disease activity, and grip strength in patients with RA. However, the balance of evidence showed that yoga had no significant effect in improving pain, tender joints, swollen joints count, and inflammatory cytokines in patients suffering from RA. Considering methodological limitations, small sample size, and low-quality, we draw a very cautious conclusion in the results of the estimate of the effect. High-quality and large-scale RCTs are urgently needed in the future, and the real result may be substantially different.

## Introduction

Rheumatoid arthritis (RA) is a chronic inflammatory autoimmune disease, characterized by pain, stiffness, swelling, loss of joint function, and elevation of acute-phase reactant levels (1). This painful chronic condition not only impairs the quality of life but also one of the leading causes of deformities or disabilities (2). Over the past decade, RA has become a major public health problem that affects nearly 1% of the world's population (3). It is more prevalent in women than in men and may occur at any age; peak incidence is between ages 50–60 years. In clinic, RA is typically treated with Disease-Modifying Antirheumatic Drugs (DMARDs), Non-Steroidal Anti-Inflammatory Drugs (NSAIDs), and Glucocorticoids (GC) (4). Unfortunately, longtime medication given for treatment have adverse effects such as immunosuppression, bone marrow dysfunction, interstitial lung disease, liver damage, hyperglycemia, and hypertension (5, 6). Thus, patients often seek more complementary therapies.

In recent years, a variety of non-pharmacologic strategies have been increasingly explored in the treatment of RA and emerged as a potential method to relieve the symptoms of RA patients (7). For example, previous literature has reported that such as cognitive behavioral therapy, physical exercise, and Complementary and Alternative Medicine (CAM) can be helpful for people with RA (8–10). As an important non-pharmacological treatment approach, yoga has received ever-growing attention in RA treatment (11). Yoga is an ancient practice that originated in India about 5,000 years ago and is reported to improve health-related quality of life, including physical function, and inflammatory symptoms in RA patients (12). Evans et al. (13) reported that yoga reduced daily pain in RA patients, but no difference in physical function. Other studies conducted on RA patients reported improvements in physical function (14), disease activity (15), grip strength (16), and a drastic reduction in the expression of inflammatory cytokines (i.e., CRP, ESR, IL-6, and TNF-α) (17).

Although according to the currently reported literature, yoga seems to be effective in the treatment of people with RA, no systematic review and meta-analysis so far have assessed its efficacy in this condition. Over the past 2 years, three randomized controlled trials (RCTs) on the effect of yoga have been published (14, 17, 18). Hence, systematic evaluation of current research is needed, and the purpose of this meta-analysis is to review and analyze the randomized effectiveness of yoga in the treatment of RA. Several variables were compared, including pain, physical function (HAQ-DI), disease activity [i.e., tender joints or swollen joints count, Disease Activity Score 28 (DAS-28)], inflammatory cytokines and grip strength.

## Methods

This study was conducted according to the PRISMA (Preferred Reporting Items for Systematic reviews and Meta-Analyses) guidelines and the recommendations of the Cochrane Collaboration in conducting this systematic review and meta-analysis (19).

### Selection Criteria

#### Studies Types

Only randomized controlled trials (RCTs) were included while observational studies or non-randomized trials were excluded. Only articles published in the English language were considered eligible.

#### Patients

Studies were required to include patients diagnosed with RA using physician-diagnosed or the American College of Rheumatology (ACR) classification criteria (20). All studies on patients with RA of the knee, hand, and feet were considered. No restrictions were applied regarding age, gender, and comorbidities, and diagnostic criteria utilized.

#### Interventions

RCTs that assessed yoga as the main intervention were included. No restrictions regarding yoga style, length, or frequency of the intervention period were applied. Differences between various types of experimental interventions were examined in subgroup analyses if applicable. When co-interventions (such as pharmacotherapy) were applied, studies were eligible only if all participants in all groups received the same co-interventions.

#### Specific Comparisons

We searched for RCTs that included one of the following group comparisons.

- Yoga intervention vs. non-yoga intervention (e.g., usual care, no intervention for control)
- Yoga intervention with medication vs. non-yoga intervention with medication.

#### Outcomes

For inclusion in this review, RCTs had to assess at least one major outcome or minor outcome:

The major outcomes included:

- Pain
- Physical function, as measured using Health Assessment Questionnaires-Disability Index (HAQ-DI)
- Disease activity, as measured using Tender joints or Swollen joints count, Disease Activity Score 28 (DAS-28)
- Inflammatory cytokines, as measured using C-reactive Protein (CRP), Erythrocyte sedimentation rate (ESR), Interleukin-6 (IL-6), Tumor necrosis factor-α (TNF-α).

The minor outcome included:

- Grip Strength.

### Search Strategy

A comprehensive search of the literature was performed by two independent reviewers (XL Ye and ZH Chen), using the following electronic databases: Medline (through PubMed), EMBASE (through SCOPUS), Cochrane Library and Web of Science database. The following search terms were used: *((Arthritis, Rheumatoid [MeSH Terms] OR Rheumatoid Arthritis [Title/Abstract]) AND [(Yoga [MeSH Terms] OR Yoga [Title/Abstract] OR Yogic [Title/Abstract]))*. In order to identify the association between yoga and rheumatoid arthritis, a filter for randomized controlled trials was added *(“Randomized Controlled Trial” [Publication Type] OR “Clinical Trial” [Publication Type] OR Tandomly [Title/Abstract] OR Randomized [Title/Abstract] OR Trial [Title/Abstract] OR Control [Title/Abstract] OR Controlled [Title/Abstract])*. All databases were searched from their inception to May 2020. Besides, we checked new literature again until May 2020, to ensure any latest articles could be included. Disagreements between two reviewers were discussed with a third reviewer (Z Shen) until consensus was reached. The search strategy is detailed in the Supplementary Appendix—search algorithm.

### Data Extraction and Quality Assessment

Two reviewers (XL Ye and ZH Chen) independently extracted study data from the eligible studies, including patient characteristics (e.g., age, gender, disease activity), study characteristics (e.g., study design, publication year, country of origin, inclusion/exclusion criteria, sample size), and study outcomes. Discrepancies were discussed with a third reviewer (Z Shen) until a consensus was achieved. The original trial authors were contacted for additional details if necessary.

### Assessment of Risk of Bias in Included Studies

The quality and risk of bias in included studies were assessed independently by 2 reviewers (XL Ye and ZH Chen) using the Cochrane risk-of-bias tool, which includes the following domains: selection bias, performance bias, detection bias, attrition bias, reporting bias, and other bias (21). Discrepancies were rechecked by a third reviewer (Z Shen) and consensus achieved by further discussion.

### Statistical Analysis

#### Assessment of Overall Effect Size

Meta-analysis was performed using Review Manager version 5.3 (Cochrane Collaboration, Oxford, UK). The mean differences (MDs), standardized mean differences (SMD), and 95% confidence intervals (CIs) for pooled data were calculated using fixed-models as outcomes were measured on the same standard scales.

#### Assessment of Subgroup

Subgroup analyses were planned according to the type of interventions (yoga with medication vs. medication, yoga vs. non-intervention and yoga vs. usual care) when sufficient studies were available and the data were heterogeneous.

#### Assessment of Heterogeneity

Statistical heterogeneity between studies was explored using *I*^2^ and chi-square statistics. *I*^2^ < 50% was regarded as no significant statistical heterogeneity and the fixed-effect model was used for analysis. When *I*^2^ > 50%, the random-effect model was used for analysis. A *P* ≤ 0.1 from Cochran's Q indicates no significant heterogeneity.

#### Assessment of Sensitivity

Owing to the small number of studies, we did not perform originally planned sensitivity analyses on inclusion/exclusion decisions, methodological quality, adequacy of the randomization process, or use of the intention-to-treat principle.

#### Assessment of Publication Bias

Publication bias was evaluated through Egger's test by using Stata version 14.0 (StataCorp LP., College Station, TX, USA).

## Result

### Study Selection

**Figure 1:**
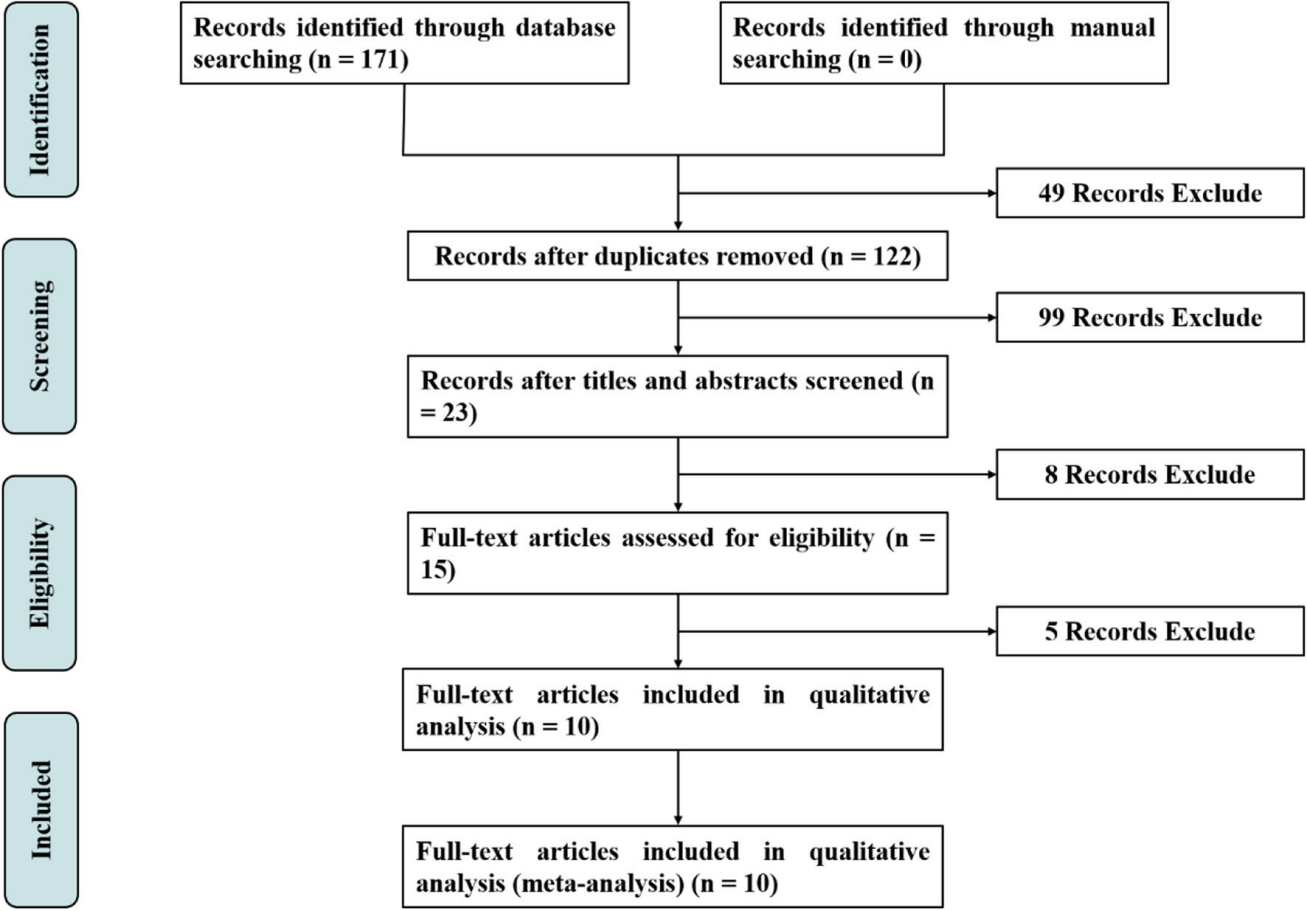
Flowchart of meta-analysis search and selection process.

The results of the literature search and selection process are summarized in Figure 1. The initial search identified 171 potentially relevant records by searching electronic databases. After the first selection, a total of 49 duplicate records were excluded. Of 122 non-duplicate records, 99 were excluded because they did not meet the inclusion criteria after going through the titles and abstracts. Of those, 23 articles were retrieved in full article form for further reading, and 8 articles were excluded for meta-analysis after reading the title and abstract. After reading the full text of the remaining 15 RCTs, 5 RCTs were considered unsuitable for the systematic review. The reasons for these excluded RCTs are shown in Supplementary Table 1. Finally, 10 RCTs reported on a total of 840 patients were included in the meta-analyses (13–18, 22–25).

### Study Characteristics

#### Overview of Included Studies

The study characteristics of the 10 RCTs are shown in Table 1. These studies were published from form 1994 to 2020. Among the 10 RCTs, four each were conducted in the United States (13, 14, 24, 25) and India (16–18, 23), and one each in England (22) and the United Arab Emirates (15). Of the 10 studies included, 840 patients with RA were analyzed. The number of enrolled patients with RA ranged from 16 to 371, and their average mean (SD) age ranging from 27.1 (4.2) to 66.7 (5.8) years. Of all 840 patients with RA in the systematic review were predominantly female (86%). Eight of the studies reported the duration of symptoms with the mean duration ranging from 6.8 (6.3) to 24.3 (11.9) years.

**Table 1:** Study characteristics.

**References**	**Population**	**Country**	**Study design**	**Mean age (SD), years**		**Sample size**		**Male/female**		**Symptom duration in years (SD)**	
				**Yoga**	**Control**	**Yoga**	**Control**	**Yoga**	**Control**	**Yoga**	**Control**
Haslock et al. (22)	RA^a^	England	RCT	52.5 (19.3)	57 (8.1)	10	10	1/9	2/8	14.8 (11.6)	19.1 (12.7)
Singh et al. (23)	RA^a^	India	RCT	34.65 (7.3)	34.65 (7.3)	40	40	13/27	11/29	11.5 (7.0)	12.1 (7.0)
Bosch et al. (25)	RA^b^	The U.S.	RCT	56.3 (7.6)	66.7 (5.8)	9	7	0/9	0/7	19.7 (6.8)	17.3 (4.4)
Evans et al. (13)	RA^b^	The U.S.	RCT	29.9 (2.9)	27.1 (4.2)	11	15	0/11	0/15	15.8 (9.8)	6.8 (6.3)
Gautam et al. (17)	RA^b^	India	RCT	45.7 (1.6)	42.1 (1.7)	36	36	7/29	9/27	6.32 (0.8)	5.61 (0.7)
Greysen et al. (14)	RA^b^	The U.S.	RCT	51.9 (13.7)	62.4 (11.9)	42	329	2/40	37/292	21.1 (12.4)	24.3 (11.9)
Badsha et al. (15)	RA^b^	The U.A.E.	RCT	44.0 (10.0)	46.2 (10.7)	26	21	0/26	0/21	9.3 (11.8)	8.2 (10)
Dash and Telles (16)	RA^a^	India	RCT	34.0 (6.5)	31.6 (6.6)	20	20	10/10	10/10	Not reported	
Moonaz et al. (24)	RA^a^	The U.S.	RCT	49.2 (13.2)	55.9 (8.9)	11	14	0/11	0/14	9.9 (8.7)	8.6 (9.4)
Ganesan et al. (18)	RA^b^	India	RCT	41.3 (9.5)	42.6 (7.1)	68	75	5/63	7/68	Not reported	

a *Physician diagnosed RA*.

b *1987 ACR RA Diagnostic Criteria*.

*U.S., United States of America; U.A.E., The United Arab Emirates; Study design: RCT, Randomized Controlled Trials; SD, Standard deviation*.

#### Intervention Characteristics and Outcome Measures

The overview of yoga intervention and outcome measures in Table 2. Regarding the interventions, half of the studies did not specify the style of yoga used (50%, *n* = 5) (13, 14, 22–24). Of those which specified the styles of yoga, 20% (*n* = 2) used Pranayama Yoga (16, 18), 10% (*n* = 1) Hatha Yoga (25), 10% (*n* = 1) Vishwas–Raj Yoga (15), and 10% (*n* = 1) of studies performed Patanjali's Raj Yoga (17). The yoga group will perform yoga intervention for at least 30 consecutive min each time with a frequency of 2–6 times a week. The length of the supervised training programs differed between 40 days and 12 weeks. In these 10 studies, six studies applied additional the same medication treatment as the control group (15, 16, 18, 22, 23, 25). Two of the six studies used disease-modifying anti-rheumatic drugs (DMARDs) in the yoga group and the control group (15, 25), one used Non-steroidal Anti-inflammatory Drugs (NSAIDs) (16), and three continued to use the previous prescription (18, 22, 23). Besides, three of the other four control groups took usual-care, which means that patients needed to perform normal physical exercise under the guidance of physicians (13, 17, 24). No intervention (no specific treatment) was acceptable as a control intervention in another study (14).

**Table 2:** Intervention, main measures, and results.

**References**	**Type of yoga practice**	**Intervention length, frequency, and duration**		**Main outcomes and result**
		**Yoga**	**Control**	
Haslock et al. (22)	Yoga (NS)	Yoga (120 min each; five weekly sessions; 12 weeks) + medication (Previous prescription)	Medication (Previous prescription)	1. Grip strength ^*^
Singh et al. (23)	Yoga (NS)	Yoga (90 min each; six weekly sessions; 40 days) + medication (Previous prescription)	Medication (Previous prescription)	1. Pain (SDPIS); 2. Inflammatory cytokines (CRP)^*^
Bosch et al. (25)	Hatha Yoga	Yoga (75 min each; three weekly sessions; 10 weeks) + medication (DMARDs)	Medication (DMARDs)	1. Physical function (HAQ-DI)^*^
Evans et al. (13)	Yoga (NS)	Yoga (90 min each; two weekly sessions; 6 weeks)	Usual-care (normal physical exercise)	1. Pain (SF-36)^*^; 2. Physical function (HAQ-DI); 3. DAS-28^*^
Gautam et al. (17)	Patanjali's Raj Yoga	Yoga (120 min each; five weekly sessions; 8 weeks)	Usual-care (normal physical exercise)	1. Physical function (HAQ-DI)^*^; 2. DAS-28^*^; 3. Inflammatory cytokines (CRP)^*^; 4. Inflammatory cytokines (ESR)^*^; 5. Inflammatory cytokines (IL-6)^*^; 6. Inflammatory cytokines (TNF-α)^*^
Greysen et al. (14)	Yoga (NS)	Yoga (120 min each; one weekly session; 12 weeks)	No intervention (no specific treatment)	1. Pain (NRS)^*^; 2. Physical function (HAQ-DI)^*^
Badsha et al. (15)	Vishwas–Raj Yoga	Yoga (60 min each; two weekly sessions; 6 weeks) + medication (DMARDs)	Medication (DMARDs)	1. Pain (SF-36); 2. Physical function (HAQ-DI)^*^; 3. DAS-28^*^; 4. Inflammatory cytokines (ESR); 5. Tender joints count^*^; 6. Swollen joints count^*^
Dash and Telles (16)	Pranayama Yoga	Yoga (60 min each; five weekly sessions; 12 weeks) + medication (NSAIDs)	Medication (NSAIDs)	1. Grip strength^*^
Moonaz et al. (24)	Yoga (NS)	Yoga (60 min each; two weekly sessions; 8 weeks)	Usual-care (normal physical exercise)	1. Tender joints count^*^; 2. Swollen joints count^*^
Ganesan et al. (18)	Pranayama Yoga	Yoga (30 min each; three weekly sessions; 12 weeks) + medication (Previous prescription)	Medication (Previous prescription)	1. DAS-28^*^; 2. Inflammatory cytokines (IL-6)^*^; 3. Inflammatory cytokines (TNF-α)^*^

*NS, not specified; Main outcome: SDPIS, Simple Descriptive Pain Intensity Scale; CRP, C-Reactive Protein; HAQ-DI, Health Assessment Questionnaire Disability Index; DAS-28, Disease Activity Score 28; ESR, Erythrocyte Sedimentation Rate; IL-6, Interleukin-6; TNF-α, Tumor necrosis factor-α; NRS, Numeric rating scale; SF-36, Short Form-36 Health Survey Questionnaire*.

* *Denotes sign. post-interventional group differences in favor of yoga group*.

The included RCTs chose somewhat different outcomes as primary outcome–Health Assessment Questionnaires-Disability Index (HAQ-DI), tender joints or swollen joints count, Disease Activity Score 28 (DAS-28), C-reactive protein (CRP), Erythrocyte sedimentation rate (ESR), Interleukin-6 (IL-6), Tumor necrosis factor-α (TNF-α). *Four* RCTs used a simple descriptive pain intensity scale (SDPIS) (23), Numeric Rating Scale (NRS) (14), and Short Form-36 Health Survey Questionnaire (SF-36) (13, 15) to measure pain in turn. Two RCTs (16, 22) measured grip strength as a minor outcome.

### Risk of Bias and Quality Assessment

The risk of bias for each study is shown in detail in Table 3. Five RCTs did not report an adequate form of randomization and the allocation concealment of these five RCTs remained unclear (14–16, 22, 25). Four RCTs (13, 15, 23, 25) were judged as unclear for performance bias. In four RCTs (16, 22–24), detection bias remained unclear. Incomplete outcome data (attrition bias) four RCTs (13, 15, 16, 25) were judged as unclear due to a lack of data detail. The risk of reporting bias was low in only two of the included RCTs (16, 22).

**Table 3:** Risk of bias assessment.

**References**	**Selection bias**		**Performance bias**	**Detection bias**	**Attrition bias**	**Reporting bias**	**Other bias**
	**Random sequence generation**	**Allocation concealment**	**Blinding of participants and personnel**	**Blinding of outcome assessment**	**Incomplete outcome data**	**Selective reporting**	
Haslock et al. (22)	Unclear	Unclear	Low	Unclear	Low	Unclear	Unclear
Singh et al. (23)	Low	Low	Unclear	Unclear	Low	Low	Unclear
Bosch et al. (25)	Unclear	Unclear	Unclear	Low	Unclear	Low	Unclear
Evans et al. (13)	Low	Low	Unclear	Low	Unclear	Low	Low
Gautam et al. (17)	Low	Low	Low	Low	Low	Low	Low
Greysen et al. (14)	Unclear	Unclear	Low	Low	Low	Low	Low
Badsha et al. (15)	Unclear	Unclear	Unclear	Low	Unclear	Low	Unclear
Dash and Telles (16)	Unclear	Unclear	Low	Unclear	Unclear	Unclear	Unclear
Moonaz et al. (24)	Low	Low	Low	Unclear	Low	Low	Low
Ganesan et al. (18)	Low	Low	Low	Low	Low	Low	Low

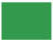
 Low risk of bias

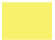
 Unclear risk of bias

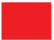
 High risk of bias

### Assessment of Overall Effect Size

#### Pain

**Figure 2:**
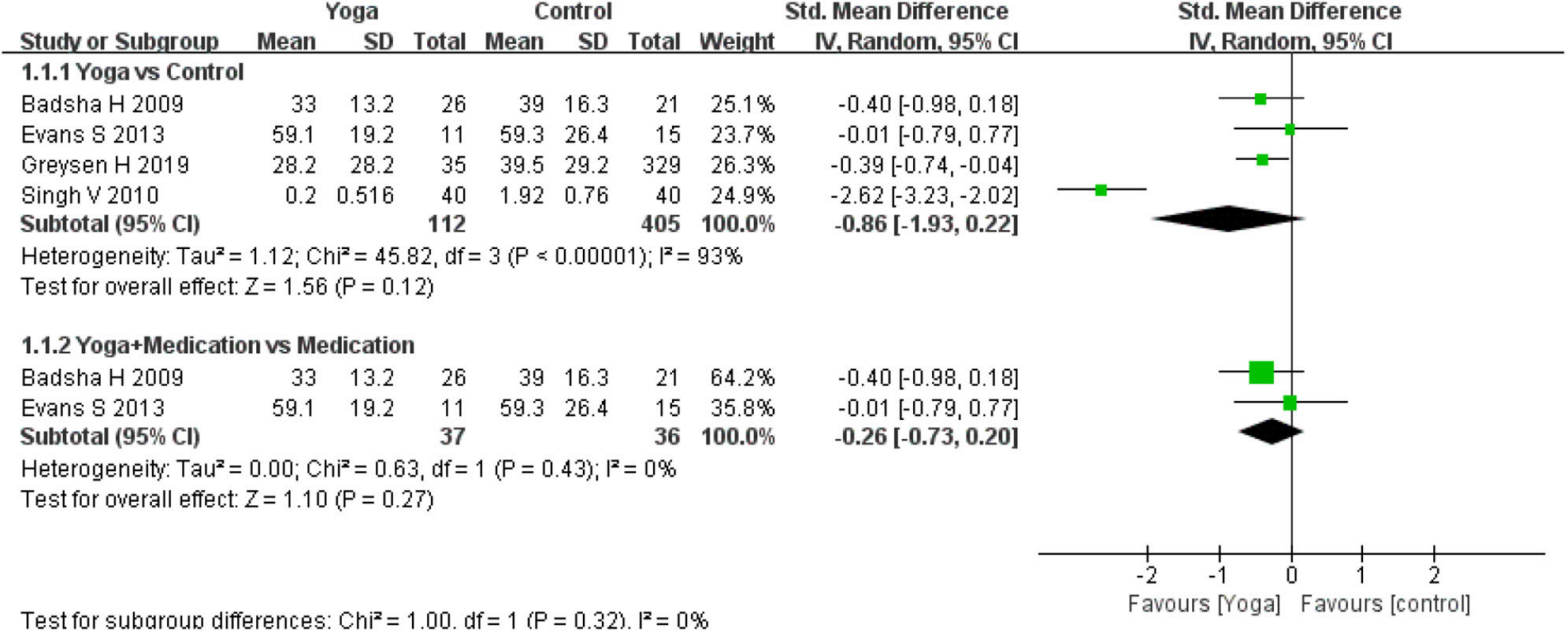
Meta-analysis on *Pain*.

Pain intensity was assessed by four of the included RCTs. Four RCTs used a Simple Descriptive Pain Intensity Scale (SDPIS) (23), Numeric Rating Scale (NRS) (14), and Short Form-36 Health Survey Questionnaire (SF-36) (13, 15) to measure pain. Higher scores of these scales denote more pain intensity. Significant effects were found for pain intensity for yoga compared to controls (4 RCTs; SMD = −1.34, 95% CI −2.49 to −0.19, *I*^2^ = 94%, *P* = 0.12). When yoga with medication is compared with medication, the subgroup analysis results revealed that there was no difference between the interventions (2 RCTs; SMD = −0.26 (95% CI: −0.73–0.20, *I*^2^ = 0%, *P* = 0.27) (Figure 2).

#### Physical Function

**Figure 3:**
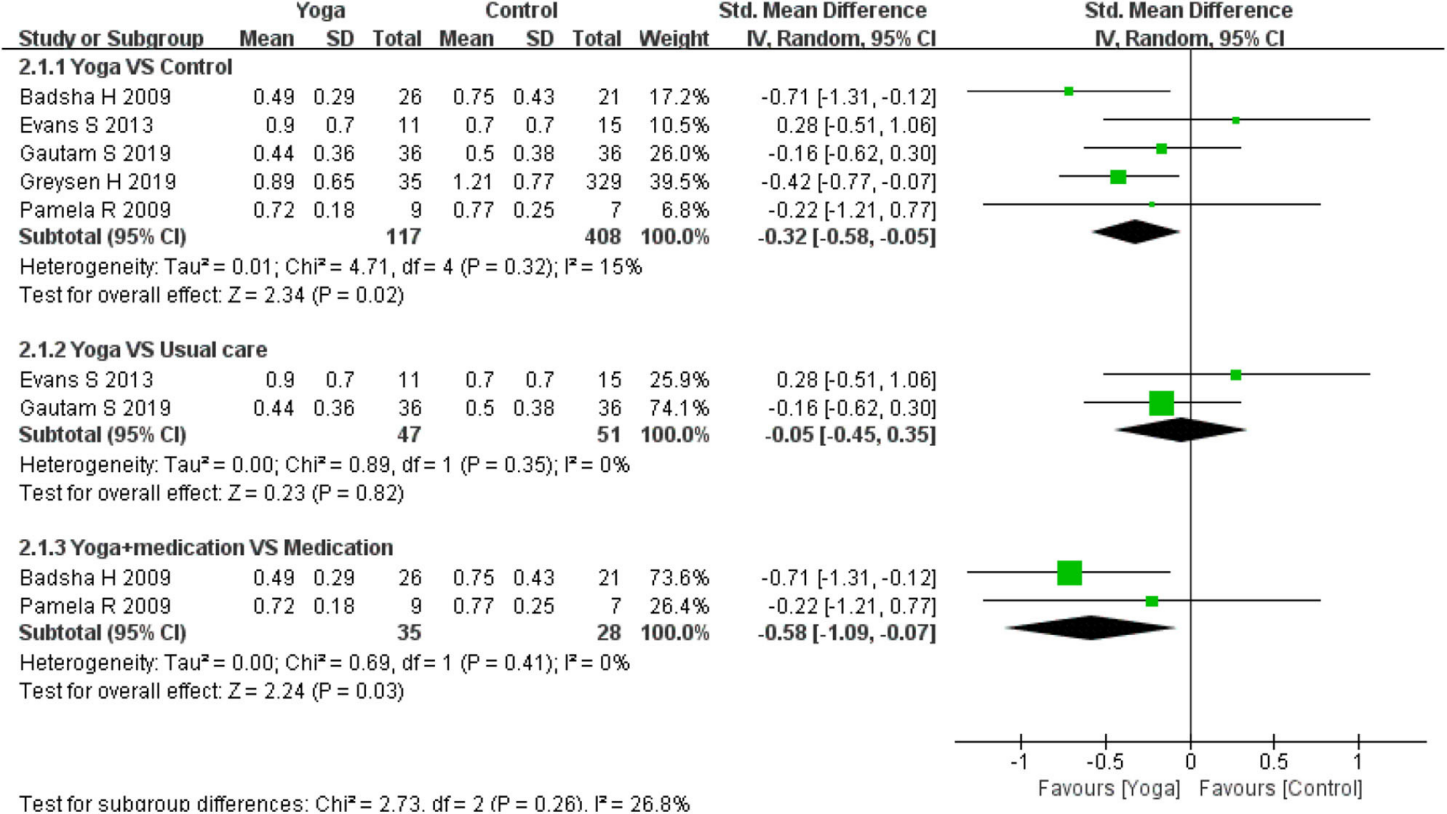
Meta-analysis on *HAQ-DI*.

Five RCTs (13–15, 17, 25) used the Health Assessment Questionnaires-Disability Index (HAQ-DI) to assess physical function. Meta-analyses generally revealed significant effect regarding physical function based on five RCTs for the comparison yoga to controls (5 RCTs; SMD = −0.32, 95% CI −0.58 to −0.05, *I*^2^ = 15%, *P* = 0.02). When comparing yoga with usual care, the subgroup analysis results revealed that there was no difference between the interventions (2 RCTs; SMD = −0.32 (95% CI: −0.45–0.35, *I*^2^ = 0%, *P* = 0.82). However, the effect for yoga with medication in comparison to medication also to be statistically significant (2 RCTs; SMD = −0.58 (95% CI: −1.09 to −0.07, *I*^2^ = 0%, *P* = 0.03) (Figure 3).

#### Disease Activity

**Figure 4:**
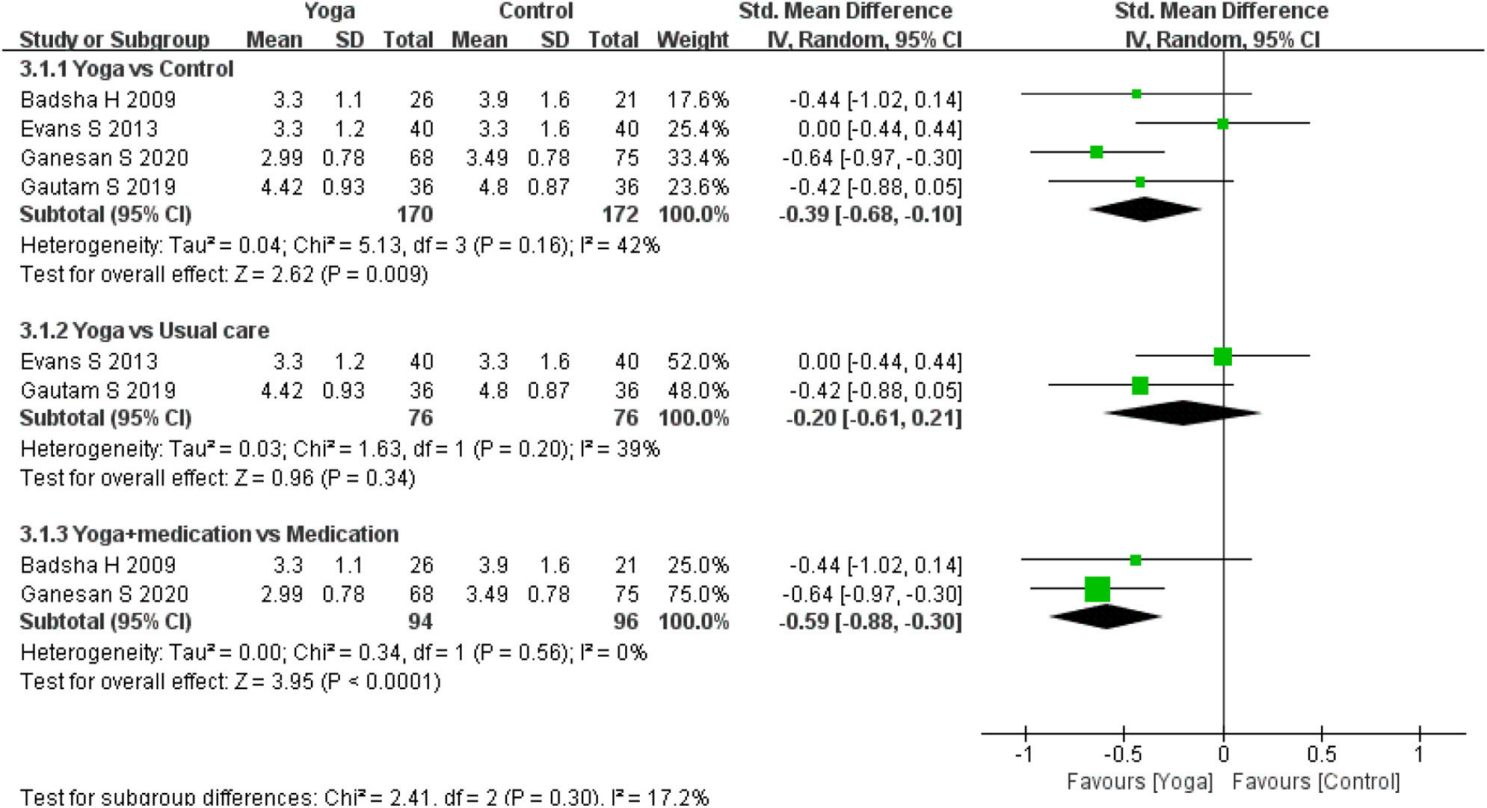
Meta-analysis on *DAS-28*.

**Figure 5:**
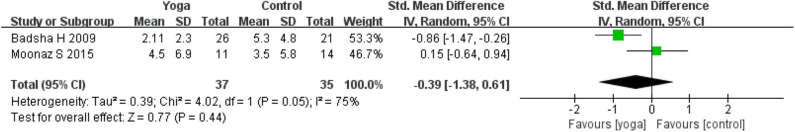
Meta-analysis on *Tender joints count*.

**Figure 6:**
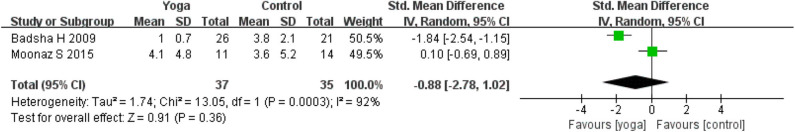
Meta-analysis on *Swollen joints count*.

Disease activity was assessed by four RCTs. Four RCTs (13, 15, 17, 18) used a Disease Activity Score 28 (DAS-28) and two RCTs (15, 24) used tender joints count and swollen joints count to assess disease activity of RA during intervention period. A statistically significant effect could be detected in DAS-28 for yoga compared to controls based on four RCTs (4 RCTs; SMD = −0.39, 95% CI −0.68 to −0.10, *I*^2^ = 42%, *P* = 0.009). Subgroup analysis shown that there was significant difference between the yoga with medication and medication group (2 RCT; SMD = −0.59, 95% CI −0.88 to −0.30, *I*^2^ = 0%, *P* < 0.0001), but no significant difference were found in yoga compares usual care group (2 RCT; SMD = −0.20, 95% CI −0.61 to −0.10, *I*^2^ = 42%, *P* = 0.34). The effects for tender joints count (2 RCTs; SMD = −0.39, 95% CI −1.38–0.61, *I*^2^ = 75%, *P* = 0.44) and swollen joints count (2 RCT; SMD = −0.88, 95% CI −2.78–1.02, *I*^2^ = 92%, *P* = 0.36) statistically turned to be no significant (Figures 4–6).

#### Inflammatory Cytokines

**Figure 7:**
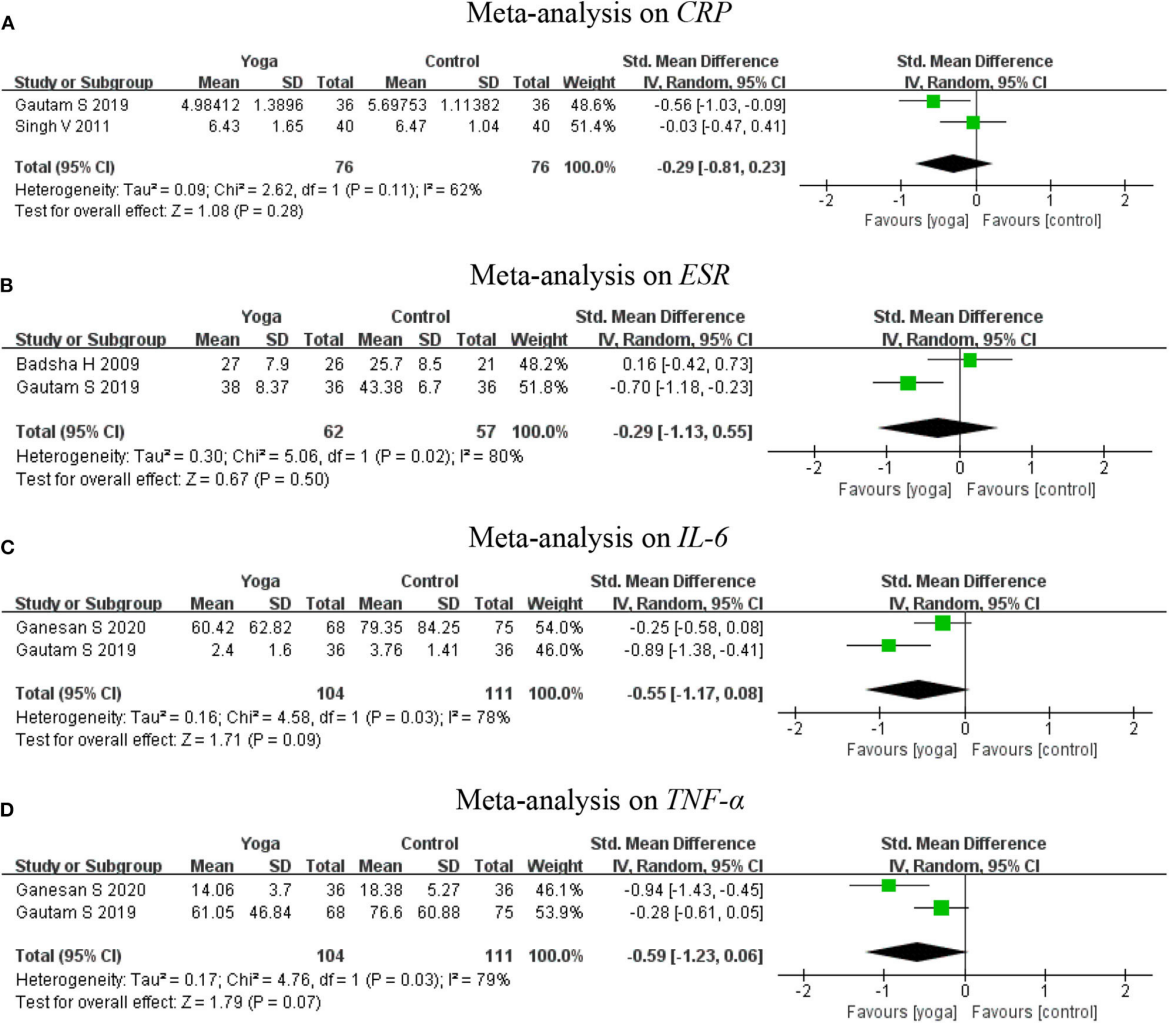
Meta-analysis on Inflammatory cytokines (**A:** *CRP*, **B:** *ESR*, **C:** *IL-6*, **D:** *TNF-*α). **(A)** Meta-analysis on *CRP*. **(B)** Meta-analysis on *ESR*. **(C)** Meta-analysis on *IL-6*. **(D)** Meta-analysis on *TNF-*α.

Inflammatory cytokines were assessed by CRP, ESR, IL-6, and TNF-α. Meta-analyses further revealed no significant effect regarding CRP based on two RCTs (17, 23) (2 RCTs; SMD = −0.29, 95% CI −0.81–0.23, *I*^2^ = 62%, *P* = 0.28), two RCTs (15, 17) for ESR (2 RCTs; SMD = −0.29, 95% CI −1.13–0.55, *I*^2^ = 80%, *P* = 0.50), two RCTs (17, 18) for IL-6 (2 RCTs; SMD = −0.55, 95% CI −1.17–0.88, *I*^2^ = 78%, *P* = 0.09) and two RCTs (17, 18) for TNF-α (2 RCTs; SMD = −0.59, 95% CI −1.23–0.06, *I*^2^ = 79%, *P* = 0.07) (Figure 7).

#### Grip Strength

**Figure 8:**
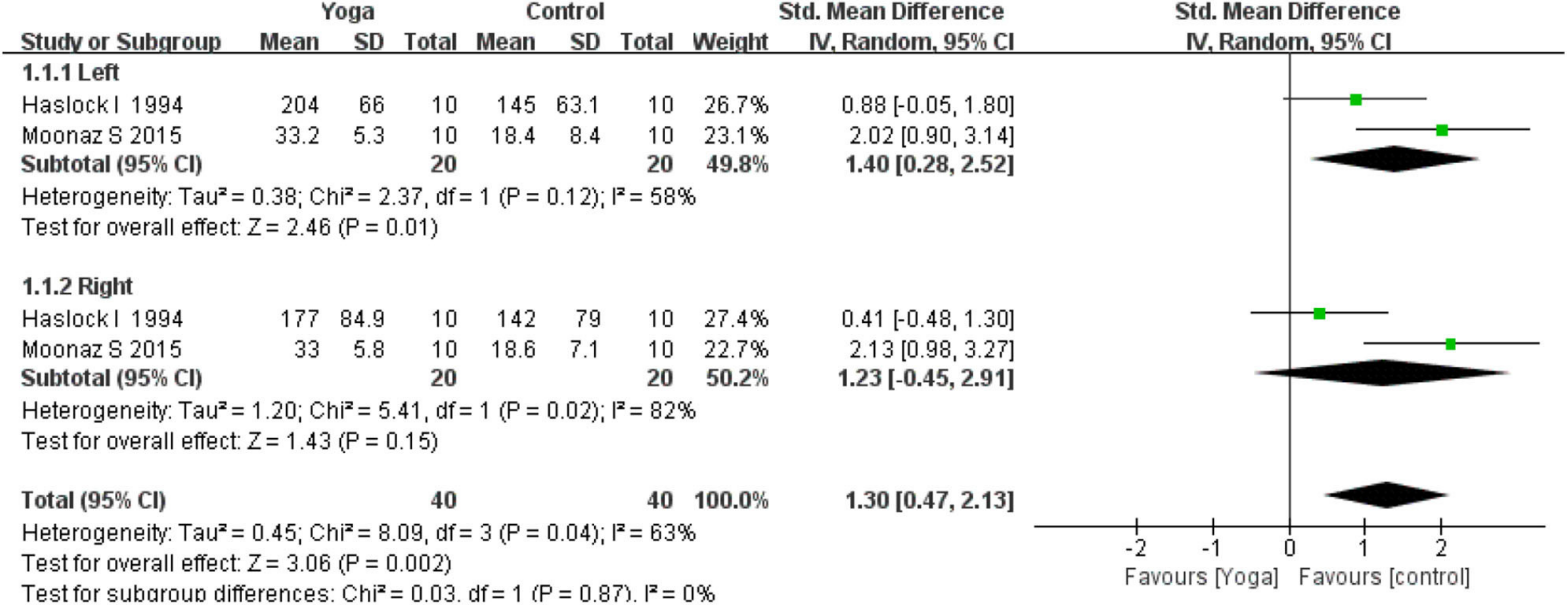
A meta-analysis on the *Grip strength*.

Subgroup analysis of grip strength was conducted based on the left and right hand. Two RCTs (16, 22) assessed the left and right grip strength. The subgroup analysis results revealed that yoga had positive effects on the grip strength of the left hand (2 RCTs; SMD = 1.40, 95% CI 0.28–2.52, *I*^2^ = 58%, *P* = 0.01), but had no significant effect on the right hand (2 RCTs; SMD = 1.23, 95% CI −0.45–2.91, *I*^2^ = 82%, *P* = 0.15). Overall, meta-analyses revealed a statistically significant effect for yoga compared to controls (2 RCTs; SMD = 1.30, 95% CI 0.47–2.13, *I*^2^ = 63%, *P* = 0.002) (Figure 8).

### Publication Bias

When the meta-analysis includes more than 10 studies with a funnel chart representing each follow-up time point, the possibility of publication bias should be reported (26). For continuous results, Egger's tests and Begg's tests should be used to assessing the possible publication bias (21, 27). However, none of the meta-analysis groups have more than 10 RCTs and therefore neither of these methods was completed.

### Safety

None of the included RCTs reported adverse events.

## Discussion

### Summary of Evidence

We conducted this review to evaluate scientific evidence for the benefits of yoga intervention compared with no interventions in people with RA. Major outcomes evaluated were pain, physical function (HAQ-DI), disease activity (tender joints or swollen joints count, DAS-28), and inflammatory cytokines (CRP, ESR, IL-6, TNF-α). Minor outcomes evaluated included grip strength. Overall results of meta-analyses suggest that performing yoga may be beneficial for improving physical function (HAQ-DI) and disease activity (DAS-28). However, considering that one of the articles has a high disease duration and a high weight, we removed it and analyzed it and found yoga has no significant effect regarding physical function (14). This shows that yoga may improve the body function of patients with a high disease duration better than patients with a relatively short duration. Of course, given the quality of the evidence is very low, we are unable to definitively state this result. Investigators also found a beneficial effect of yoga on grip strength, but heterogeneity between studies was greater and the confidence interval was slightly larger. Evidence was insufficient to show the effect of yoga on pain, disease activity (tender joints or swollen joints count) and inflammatory cytokines in patients with RA.

### Comparison to Prior Reviews

To the best of our knowledge, this is the first systematic review and meta-analysis to evaluate the effectiveness of yoga in RA. Several years ago, one review included eight studies has examined the effects of yoga on rheumatic conditions, and the authors concluded that yoga is a useful add-on therapy for treating RA and the most significant benefits of yoga was practiced in a combination of physical postures, regulated breathing, meditation, and yoga philosophy (28). While the authors of this previous review cannot perform a meta-analysis, the increased number of publications in recent years allowed for a meta-analysis at this point, and effect estimates could be provided. Overall, our meta-analysis found that yoga may improve the physical function (HAQ-DI) and disease activity (DAS-28) for patients with RA, and the meta-analyses showed no differences between the yoga and non-exercise controls including usual care. However, the results found are inconsistent with previous literature reports.

### Is Yoga an Effective Treatment for Rheumatoid Arthritis?

Although the cause of RA remains unknown and incurable, controlling symptoms, preventing joint damage, and maintaining physical function to improving the RA patient's quality of life is the main target of treatment currently (29). And balancing and resolving inflammation is key to optimal therapeutic success (30). Non-pharmacological interventions are believed beneficial for RA, and as the American College of Rheumatology (ACR) points out that stretching, strengthening, and conditioning exercise can maintain physical function (31). The practice of yoga involves specific physical postures (asanas), breath regulation (pranayamas), concentration (dharana), and meditation (dhyana) (32). Yoga is reported to improve people with RA physical and mental health, including physical function and depressive symptoms, pain, and fatigue, and sleep, by incorporating meditative breathing with physical exercise (33). Yoga emphasizes breathing and relaxation exercises as well as incorporating meditation to reduce chronic pain symptoms. These exercises may stimulate the parasympathetic nervous system and help break the pressure-pain cycle to relieve chronic pain symptoms (34, 35). Additionally, the effect of yoga on the musculoskeletal system may be important for maintaining the physical function of RA. Yoga includes a variety of postures similar to static stretching, which might improve muscle strength, especially leg press strength (36, 37). Improvements in muscle function also bring the improvement of the physical function of RA. Finally, considering the important role of inflammation in the progress of RA, we analyzed the inflammation markers of patients with RA, such as CRP, ESR, IL-6, and TNF-α, which are considered the prime cytokines that reflect or drive the inflammatory process in RA (38). However, no significant effects were found in this meta-analysis, which might be due to the small sample size.

### Implications for Practice

Currently, this meta-analysis suggests that yoga will help to improve physical function and disease activity among people with RA. Therefore, a weak recommendation can be given for using yoga as a treatment strategy for RA. However, considering yoga as a non-pharmacological and low-risk intervention, it is still recommended for RA patients who do not adhere to physical exercise regularly. The future meta-analysis of high-quality studies and large sample sizes is very likely to have an important impact on our confidence in the estimates of effect and may change the estimates.

### Limitations and Quality of Evidence

However, the result of this systematic review had several limitations. Firstly, all included RCTs were from methodological limitations. Only half of the RCTs reported adequate randomization, while allocation concealment of these RCTs had to be suspected as biased. Some included RCTs also had performance, detection, attrition, and reporting bias. Therefore, the quality of evidence for outcomes had been reduced to very low levels. Secondly, only a small sample size was included. During the follow-up analysis, some meta-analyses including only 2 RCTs. Although a meta-analysis could be conducted through 2 RCTs, the conclusions drawn from the results should be considered preliminary and might be biased (39). Thirdly, the RCTs varied in terms of their patient populations (such as age and symptom duration), interventions (medication or not), comparison treatments (type, length, and frequency and duration), and outcome measures. Given the small number of trials, we were unable to examine whether the effects on the study outcomes were influenced by these factors. And these might eventually cause heterogeneity and interpretability in the results.

### Implications for Further Research

While we acknowledge the difficulty of conducting properly randomized controlled trials in this subject, ideally the next step in this subject would be to conduct studies with more robust designs as followed:

Firstly, future reporting of yoga RCTs should strictly follow standard reporting guidelines, such as CONSORT (Consolidated Standards of Reporting Trials) statement (40) to improve the quality of their research.

Secondly, prospective registration in the clinical trial registry before recruiting patients will help reduce selective reports. Researchers are recommended to disclose the complete research protocol in all relevant publications.

Thirdly, to determine the long-term impact, follow-up assessments are urgently needed at different periods.

Fourthly, none of the included RCTs reported adverse events, considering that safety is crucial for the evaluation of therapies, future RCTs should consider safety.

Fifthly, future RCTs should directly compare yoga with other interventions (such as medication, usual care, no specific treatment or any other intervention) to investigate the potential benefits of yoga in the treatment of RA patients as described above.

## Conclusion

Despite several limitations, this systematic review found preliminary evidence that yoga interventions were associated with improvement in physical function, disease activity and grip strength compared with non-yoga patients with RA. No effects were found for patients suffering from RA. Due to the methodological limitations, small sample size, and a lack of data, we can only make a weak recommendation that yoga can improve the physical function and disease activity of patients with RA. However, additional high-quality and large RCTs are needed to confirm the effectiveness of yoga interventions for the management of patients with RA.

## Data Availability Statement

The original contributions presented in the study are included in the article/Supplementary Materials, further inquiries can be directed to the corresponding authors.

## Author Contributions

XY conceived the study design. XY and ZC conducted the literature search, did some of the data extraction, did all of the analyses, and drafted the manuscript. ZS helped with the data analysis, reviewed the manuscript for important intellectual content, and made major revisions to the manuscript. XX and GC supervised the study. All authors have read and approved the final version of the submitted version.

## Conflict of Interest

The authors declare that the research was conducted in the absence of any commercial or financial relationships that could be construed as a potential conflict of interest.
