# Supplementary material for: Yoga for Treating Rheumatoid Arthritis: A Systematic Review and Meta-Analysis
Source: Front Med (Lausanne). 2020 Nov 27;7:586665. doi: 10.3389/fmed.2020.586665 (PMC7732597; doi:10.3389/fmed.2020.586665)
Supplement: Supplementary file 1 [file Table_1.DOCX]

Supporting Information

**Yoga for Treating Rheumatoid Arthritis: a Systematic Review and Meta-analysis**

Xiangling Ye ^1^ *, Zehua Chen ^1^ *, Zhen Shen ^1^ *, Guocai Chen^2 #^, Xuemeng Xu ^3 #^

*1 The Fifth Clinical Medical College, Guangzhou University of Chinese Medicine, Guangzhou, Guangdong, China, 510405.*

*2 Foshan hospital of Traditional Chinese Medicine, Foshan, Guangdong, China, 528000.*

*3 Guangdong Second Traditional Chinese Medicine Hospital, Guangzhou, Guangdong, China, 510095*

**Corresponding author at** Guangdong Second Traditional Chinese Medicine Hospital, Guangzhou, Guangdong, China, 510095.

**E-mail address:** Xuemeng Xu (xuxuemeng@163.com); Guocai Chen (chenguocai888@qq.com).

* These authors have contributed equally to this work.

**Supplementary Appendix -** **Search algorithm**

**PUBMED:**

*#1 (**Arthritis, Rheumatoid [MeSH Terms] OR Rheumatoid Arthritis[Title/Abstract])*

*147,792*

*#2 (Yoga[MeSH Terms] OR Yoga[Title/Abstract] OR Yogic[Title/Abstract]) 5,293*

*#3 #1 AND #2 47*

*#4 (“Randomized Controlled Trial”[Publication Type] OR “Clinical Trial”[Publication Type] OR Randomly[Title/Abstract] OR Randomised[Title/ Abstract] OR Trial[Title/Abstract] OR Control[Title/Abstract] OR Controlled[Title/Abstract]) 4,402,480*

*#5 #3 AND #4 26*

**EMBASE:**

*#1 ' Rheumatoid Arthritis '/exp 140,277*

*#2 ' Rheumatoid Arthritis '/ Title/Abstract 164,937*

*#3 #1 OR #2 199,518*

*#4 ' Yoga '/exp 3,549*

*#5 ' Yoga '/ Title/Abstract 6,807*

*#6 ' Yogic '/ Title/Abstract 521*

*#7 #4 OR #5 OR #6 7,208*

*#8 ' Randomized Controlled Trial '/exp 609,406*

*#9 #3 AND #7 AND #8 43*

**Cochrane Library:**

*#1 ' Rheumatoid Arthritis ' 15,701*

*#2 ' Yoga ' 3,437*

*#3 ' Yogic ' 269*

*#4 #2 OR #3 3,508*

*#5 #1 AND #4 70*

**Web of Science:**

*#1 TS = Rheumatoid Arthritis 173,483*

*#2 TI = Rheumatoid Arthritis 88,162*

*#3 #1 OR #2 173,483*

*#4 TS = Yoga 6,232*

*#5 TI = Yoga 3,728*

*#6 TI = Yogic 185*

*#7 #4 OR #5 OR #6 6,353*

*#8 TS = Randomized Controlled Trial 412,436*

*#9 #3 AND #7 AND #8 32*

**Supplementary Table 1** Excluded RCTs found from search strategy and reason

| **sTable 1 -** Excluded RCTs | | |
| --- | --- | --- |
| **Reference, Year** | **Excluded RCTs** | **Reason** |
| Evens S 2011[1] | A randomized controlled trial examining Iyengar yoga for young adults with rheumatoid arthritis: a study protocol | Protocol |
| Daniel 2014[2] | Yoga for pain and sleep quality in rheumatoid arthritis: study protocol for a pilot randomized controlled trial | Protocol |
| Mahidashtizad S 2013[3] | AB1425-HPREffects of a yoga program on pain relief, reducing anxiety and extended range of motion of knee in rheumatoid arthritis patients | Full text  unavailable |
| Middleton K[4] | A pilot study of yoga as self-care for arthritis in minority communities | Protocol |
| Ward L 2018 [5] | Yoga for the management of pain and sleep in rheumatoid arthritis: a pilot randomized controlled trial | No suitable control group |

[1] S. Evans, L. Cousins, J. Tsao, S. Subramanian, B. Sternlieb, and L.J.T. Zeltzer, A randomized controlled trial examining Iyengar yoga for young adults with rheumatoid arthritis: a study protocol. 12 (2011) 19.

[2] Daniel, Cherkin, Simon, Stebbings, G., David, Baxter, Lesley, Ward, and J.J.P.T.R. Ptr, Yoga for pain and sleep quality in rheumatoid arthritis: study protocol for a pilot randomized controlled trial. (2014).

[3] S. Mahidashtizad, and A.J.A.o.t.R.D. Salajegheh, AB1425-HPREffects of a yoga program on pain relief, reducing anxiety and extended range of motion of knee in rheumatoid arthritis patients. 71 (2013) 756-756.

[4] K. Middleton, M. Ward, S. Haaz, S. Velummylum, A. Fike, A. Acevedo, G. Tataw-Ayuketah, L. Dietz, B. Mittleman, G.J.H. Wallen, and q.o.l. outcomes, A pilot study of yoga as self-care for arthritis in minority communities. 11 (2013) 55.

[5] L. Ward, S. Stebbings, J. Athens, D. Cherkin, and G.J.M.c. David Baxter, Yoga for the management of pain and sleep in rheumatoid arthritis: a pilot randomized controlled trial. 16 (2018) 39-47.
